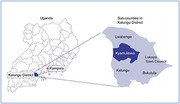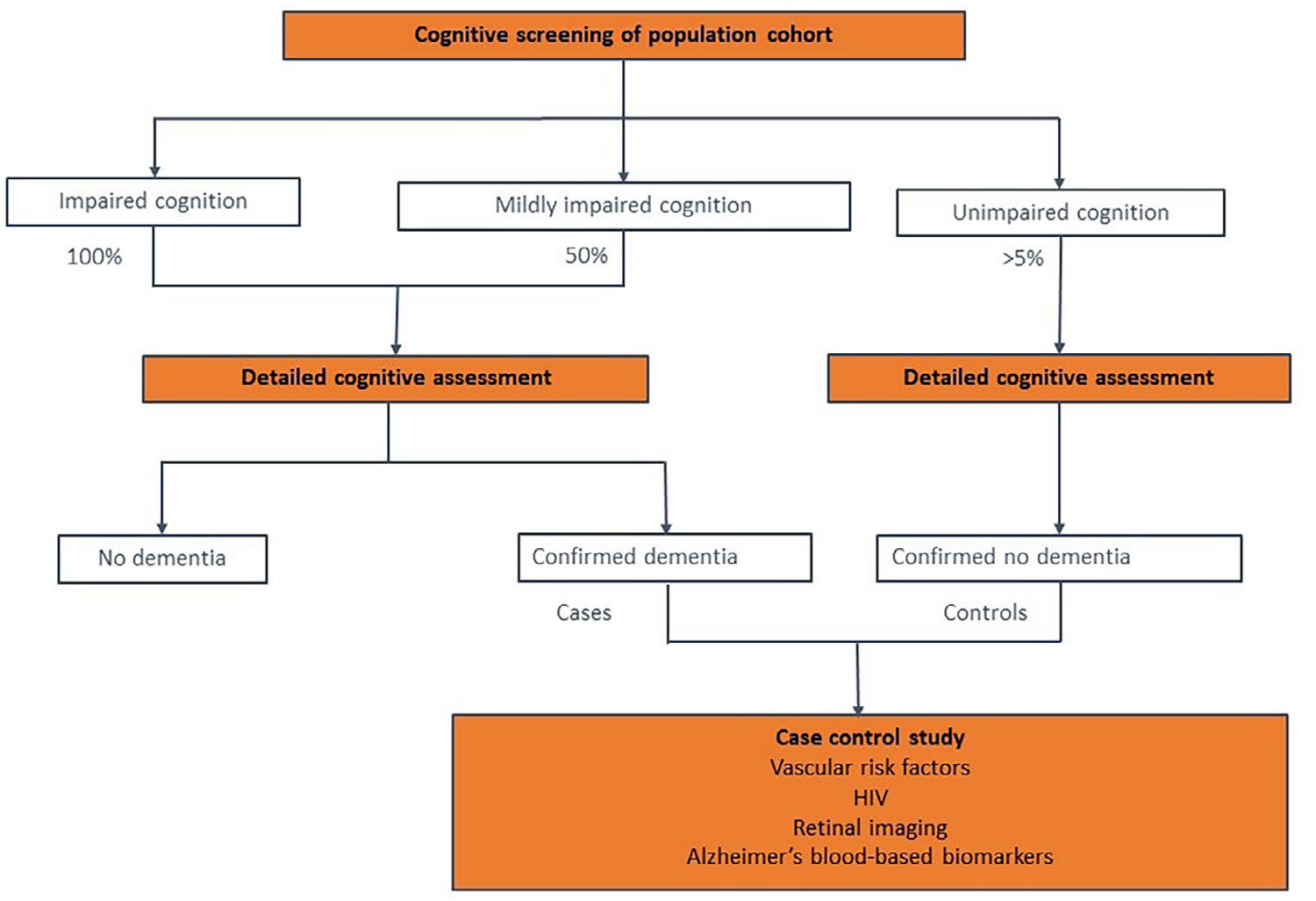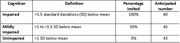# Understanding dementia aetiology in Uganda

**DOI:** 10.1002/alz.083650

**Published:** 2025-01-09

**Authors:** Josephine E Prynn, Joseph Mugisha, Claire J Steves, Tunde Peto, Beatrice Kimono, Martin J Prince

**Affiliations:** ^1^ King’s College London, Strand, London United Kingdom; ^2^ MRC/UVRI & LSHTM Uganda Research Unit, Entebbe, Wakiso Uganda; ^3^ Queen’s University Belfast, Belfast United Kingdom

## Abstract

**Background:**

The prevalence of dementia in low‐ and middle‐income countries is increasing, yet epidemiological data from African populations remains scarce. Crucial risk factors differ in Africa from more intensively studied global areas, including a high burden of cerebrovascular disease (evidenced by high stroke incidence) and HIV, but lower rates of other risk factors such as physical inactivity. In the pre‐antiretroviral therapy era, dementia was a common consequence of HIV infection. However, it is not clear from existing literature what effect, if any, chronic and well‐controlled HIV has on cognition with ageing.

Understanding dementia aetiology in African settings has been limited by the expensive and invasive nature of biomarker testing. This study leverages developments in biomarker technology to examine the drivers of dementia in older Ugandans. This includes using blood‐based biomarkers for Alzheimer’s disease and retinal imaging with automated analysis of vascular morphology as a proxy for cerebrovascular disease.

Among older adults in a Ugandan population, this study will evaluate the prevalence of dementia, establish the pathological processes underlying dementia to inform risk reduction strategies, and examine the impact of dementia on individuals and their caregivers.

**Methods:**

The study is nested within the existing General Population Cohort (GPC) run by the Medical Research Council /Uganda Virus Research Institute (MRC/UVRI) & London School of Hygiene and Tropical Medicine (LSHTM) Research Unit and includes all adults over 60.

In Step 1, cohort participants will undergo cognitive screening. Based on screening scores, a subgroup will undergo cognitive assessments using tools developed by the 10/66 Dementia Research Group to determine dementia diagnoses.

Step 2 is a case control study of people with and without dementia using antecedent data, questionnaires, physical assessment, retinal imaging, and Alzheimer’s blood‐based biomarkers. We will also compare disability, frailty, quality of life, and social engagement in people with and without dementia.

**Results:**

We will identify the prevalence of dementia, odds ratios, and population attributable fractions for both modifiable risk factors for dementia and the pathological processes of Alzheimer’s pathology and cerebrovascular disease.

**Conclusions:**

This will be first study in Africa to examine pathological processes leading to dementia including markers of Alzheimer’s and cerebrovascular disease.